# Altered oculomotor flexibility is linked to high autistic traits

**DOI:** 10.1038/s41598-023-40044-5

**Published:** 2023-08-10

**Authors:** Antonella Pomè, Sandra Tyralla, Eckart Zimmermann

**Affiliations:** Institute for Experimental Psychology, Heinrich Heine University Duesseldorf, Universitätsstr. 1, 40225 Düsseldorf, Germany

**Keywords:** Cognitive neuroscience, Sensorimotor processing, Sensory processing, Oculomotor system, Saccades, Autism spectrum disorders

## Abstract

Autism is a multifaced disorder comprising sensory abnormalities and a general inflexibility in the motor domain. The sensorimotor system is continuously challenged to answer whether motion-contingent errors result from own movements or whether they are due to external motion. Disturbances in this decision could lead to the perception of motion when there is none and to an inflexibility with regard to motor learning. Here, we test the hypothesis that altered processing of gaze-contingent sensations are responsible for both the motor inflexibility and the sensory overload in autism. We measured motor flexibility by testing how strong participants adapted in a classical saccade adaptation task. We asked healthy participants, scored for autistic traits, to make saccades to a target that was displaced either in inward or in outward direction during saccade execution. The amount of saccade adaptation, that requires to shift the internal target representation, varied with the autistic symptom severity. The higher participants scored for autistic traits, the less they adapted. In order to test for visual stability, we asked participants to localize the position of the saccade target after they completed their saccade. We found the often-reported saccade-induced mis-localization in low Autistic Quotient (AQ) participants. However, we also found mislocalization in high AQ participants despite the absence of saccade adaptation. Our data suggest that high autistic traits are associated with an oculomotor inflexibility that might produce altered processing of trans-saccadic vision which might increase the perceptual overstimulation that is experienced in autism spectrum disorders (ASD).

## Introduction

Autism spectrum disorder (ASD) is traditionally conceived as a social communication disorder, but recently it has been recognized as a complex, multisystem disorder with multiple core and comorbid impairments, including restrictive and repetitive behaviors which are present in the early developmental period^1,2^. It has been repeatedly shown that individuals with ASD struggle with making predictions or with deploying attention based on prior experiences^3–6^. Together with sensory abnormalities, a general inflexibility in the motor domain has been reported^7,8^.

Here we tested the hypothesis that motor inflexibility generates or at least contributes to the experience of an overload in sensory stimulation in high autistic symptomatology. Every movement produces a motor error and the brain must solve the credit assignment problem: did the error occur due to inaccurate movement targeting or due to a change in the external world?^9–11^ Solving this problem is particularly pressing for saccade eye movements^12^. Since saccades are made with a comparably high frequency—about three times per second—deciding whether motion on the retina was produced by the self-produced saccade or by external stimulation is of crucial importance. To counteract the visual motion on the retina generated by a saccade, an efference copy—which is a copy of the motor command—needs to be conveyed to visual areas before execution of the saccade^13–16^. The information about the saccade vector allows to predict the size and direction of the retinal motion. Comparing predicted against actual retinal motion allows to disentangle whether the retinal motion was due to a change in the external world or was produced by self-motion. Solving this ambiguity accurately is of fundamental importance for perception and action likewise. A preference towards attributing motor errors to the external world, would present a world that is ambiguous during saccades. Perceiving intra-saccadic motion, especially if it does not represent actual motion in the external world, will represent a disturbing experience. In the motor domain, attributing the causes of errors to the external world will lead to inflexibility. If a saccade fails to reach an intended target, oculomotor learning adjusts saccade control such that the following saccades minimize the targeting error^17,18^. However, if the sensorimotor system externalizes the cause of the error, no adaptation of the movement trajectories will ensue. In this view, sensory overload through intra-saccadic motion perception and motor inflexibility might result from a biased causal inference in motor error attribution.

Oculomotor learning can be studied in the laboratory by displacing the saccade target during the execution of the saccade^19,20^: the oculomotor system will adjust saccade amplitudes to minimize the post-saccadic error. Decades of research on saccade adaptation have led to an understanding of the mechanisms involved in saccade gain change^21–25^. Inward and outward gain adaptation, i.e. saccade amplitude shortening and lengthening, involve different neural mechanisms. Outward adaptation has been shown to act on a supra-retinal coordinate frame^26,27^ whereas inward adaptation operates on a general gain reduction^25,26,28–30^. Several studies have shown that outward adaptation takes more time to develop and is less complete than inward adaptation^31–33^ as well as in modifications of motor parameters^21,29,34,35^. Transfer of reactive saccade adaptation to antisaccades^36^, to hand-pointing movements^37^, and to visual perception^35^ was found only for outward but not for inward adaptation.

An explanation for these robust differences between inward and outward adaptation has been offered by optimal control models, which suggested that gain inward adaptation occurs in the forward model of saccade trajectories, whereas gain outward adaptation results from a shift in the target representation (see^38^). It has also been suggested that inward gain adaptation results from uncompensated fatigue at a cognitive level^39^.

Motor errors are processed by the sensorimotor system not only to recalibrate motor but also visual space^35,40–46^. When, after saccade adaptation, participants are asked to localize an object, their spatial estimate is shifted in the direction of adaptation. We have recently shown serial dependencies between saccadic and visual localization that recalibrate visual space according to the experienced post-saccadic errors^47^.

If autistics solve the credit assignment problem with a bias towards inferring more often motion in the external world, they should show less oculomotor adaptation, predominantly outward which requires a shift in the saccade target coordinates^12,34^. Since saccade adaptation induces the mislocalization, reduced adaptation in high autistic traits should also be accompanied by a decrease in adaptive shifts in visual localization. We performed a second experiment in which the saccade target had to be localized across the execution of a saccade. Because vision is suppressed during saccades, observers will not perceive the displacement transient and correct localization depends on a comparison of the target position before and after the saccades. Such a comparison requires an estimate about the own motor error created by the saccade. If motor errors are not interpreted as such, then no stimulus is driving the sensorimotor system to invest into movement gain change. Following this hypothesis, participants scoring high on the spectrum should indicate the target closer to the displaced position.

## Results

**Figure 1:**
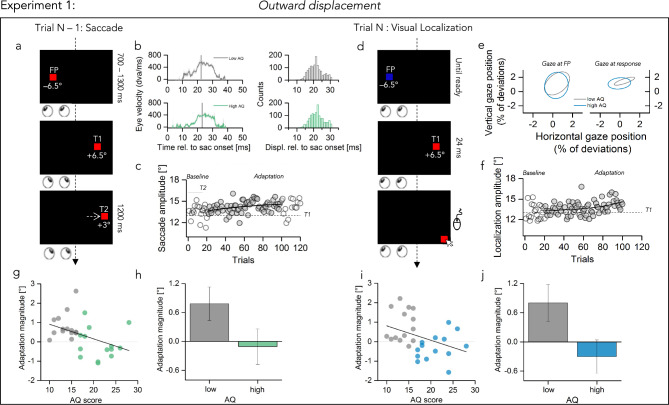
Experimental paradigm and main results of outward adaptation condition (exp1). (**a**) Schematic illustration of a saccade trial. A red fixation square (FP) was presented 6.5° left to the screen center for 700–1300 ms. A red target (T1), presented 13° apart, signaled the start of a saccade. Participants had to redirect their gaze to T1 once FP was extinguished. Once a saccade had been detected T1 was displaced 3° to its right (T2, outward displacement) and stayed on screen for 1.2 s. (**b**) Averaged eye velocity profiles representing saccade performance in the saccade trials for low AQ (in gray) and high AQ (in green). Average timing of the actual target displacement is shown by the black vertical line. The distribution of the target jumps relative to saccade onset are shown in the right small panel, separately for low and high AQ. (**c**) Saccade amplitudes over the course of the session for one example participant. Empty light gray circles represent saccade amplitudes at baseline (0–20 trials) and in de-adaptation (100–120 trials). Data outside this range (20–100) were fitted with an exponential function which best described the data. Dashed line at 13° indicate the location of T1. Amplitudes increase over the course of the trials in the direction of the target displacement (T2, black arrow at 16°). (**d**) Schematic illustration of a visual localization trial. A blue fixation square (FP) presented at the same eccentricity of the saccade FP signaled the start of a visual localization trial. Participants were instructed to fixate at that location and press the space bar when ready to start the trial. A red target (T1) was briefly flashed for 24 ms at the same eccentricity of saccade target T1 (+ 6.5° from screen center). The target had to be localized, without moving the eyes from the invisible FP location, by using a mouse cursor which appeared at the very down right border of the screen resembling the target. (**e**) Variability of gaze position, measured as the Bivariate Confidence Ellipse (95% CI) of horizontal and vertical gaze position samples (percentage of imaged area), computed after pooling data from all participants in each group and separately for gaze during fixation and gaze during response; this is similar in low and high AQ (gray and blue lines respectively), as well as during fixation (left panel) and at response (right panel). (**f**) Localization amplitudes over the course of the session for one example participant. Empty light gray circles represent localization amplitudes at baseline (0–20 trials). Data outside this range (20–100) were fitted with an exponential function which best described the data. Amplitudes increase over the course of the trials following the saccade amplitudes trend. Dashed line at 13° indicate the location of T1. (**g**) Adaptation magnitudes in degree as a function of autism severity, calculated by subtracting the first 20 trials of baseline from the last 20 trials of saccade adaptation and then averaged across participants. Each data point represents one participant (low AQ in gray and high AQ in green). Dashed line at 0 indicate no effect of target displacement, positive values indicate a shift of the effect in the direction of the displacement. Thick black line shows the linear fit through the data. (**h**) Adaptation magnitude averaged over the two groups of participants (low AQ gray, high AQ green). Bars represent average across participants (N = 15 low AQ participants, N = 13 high AQ participants), error bars show 1 SEM. (**i**,**j**) Same as (**g**,**h**) for visual localization data, color coded as gray for low AQ and light blue for high AQ. Adaptation magnitudes was calculated by subtracting the first 20 trials of baseline from the last 20 trials of localization adaptation and then averaged across participants.

We asked participants to make a rightward saccade to a target that was presented as soon as the fixation point disappeared (Fig. 1a). During the execution of the saccade, the target was—unbeknownst of the participant—displaced by 3° outward or inward depending on the condition tested. Participants tested were covering a wide range of autistic traits. The Autistic Quotient scores as measured by the Autistic Quotient questionnaire^48–50^ in our sample ranged from 10 to 28 with a mean (± SD) of 17.1 (± 4.6). The autistic traits of the whole population form a continuum, with ASD diagnosis usually situated on the high end^49,51,52^. Moreover, autistic traits share genetic and biological etiology with ASD^53^. Thus, quantifying autistic-trait-related differences in healthy people can provide unique perspectives as well as a useful surrogate for understanding the symptoms of ASD^49,54^.

Figure 1a shows the time-course of events for sessions in which the target was shifted outward as soon as the eye-tracker detected saccade initiation. We checked the timing of the grating displacement relative to saccade onset in an offline analysis. For both sub-groups (Fig. 1b), displacements were presented well within the period of saccade execution: on average 21.81 ms (± 1.88) after the saccade onset for low autistic traits—average saccade duration approximatively 45.33 ms (± 6.9)—and 22.64 ms (± 1.83) for high autistic traits—average saccade duration approximatively 41.37 ms (± 3.8). Figure 1c shows the saccadic amplitudes for one example participant. Data from the adaptation trials were fitted with an exponential function, which described the increase in saccade amplitudes caused by the displacement, over the course of adaptation ($y=14.8-1.8{e}^{-0.01x}$). After every saccade trial, a visual localization trial was presented. In these, participants were asked to localize a briefly flashed target with a mouse pointer while keeping gaze directed to the fixation point (Fig. 1d). The localization target was presented at the same eccentricity of the first saccade target (6.5° to the right of the screen center). We excluded the confounding influence of visual landmarks by conducting the experiment in a completely dark room. Gaze positions that exceeded a radius of 2.5° around the fixation point, during fixation as well as during the response period, were excluded from the analyses. Eye-movement during fixation and during the mouse response were checked to make sure both groups adequately kept ocular fixation (Fig. 1e). The mean fixation locations resulted very similar between the two groups, both before saccade execution (gaze at fixation mean ± 1 SEM: low AQ = 0.43(0.15), high AQ = 0.37(0.14), t(26) = 0.22, *p* = 0.82, log_10_BF = − 0.7) and during response period (gaze at response mean ± 1 SEM: low AQ = 1.32(0.48), high AQ = 0.24(0.16), t(26) = 1.7, *p* = 0.10, log_10_BF = − 0.13).

The dispersion of eye positions, calculated as the bivariate confidence ellipse area (BCEA), resulted similar for low and high Autistic Quotient (AQ) for both gaze at fixation (t(26) = 0.84, *p* = 0.41 , log_10_BF = –0.34) and gaze at response (t(26) = 1.76, *p* = 0.17, log_10_BF = 0.04). As for saccade trials, data outside the baseline range (up to 20 trials) were fitted whit the best exponential fit ($y=13.2+0.06{e}^{0.03x}$), which resembled the course of saccade amplitude lengthened (Fig. 1f).

We first focused on adaptive saccade flexibility and the consequences on the perception of space. In order to quantify the magnitude of this relationship, we calculated the change in saccade and visual localization by subtracting the mean saccade and localization amplitudes in the last 20 trials of the sessions from the first 20 trials of baseline for each participant. Figure 1g plots the amount of saccade adaptation for outward displacements for each participant as a function of the severity of autistic symptoms. Stepping the target 3° outward, led to recalibration of saccade amplitudes that were negatively correlated to the autistic symptomatology (r = − 0.40, p = 0.03, log_10_BF = 0.14). While people with lower degrees of autistic traits successfully used their post-saccadic error to recalibrate their motor space, the high AQ subsample failed to flexibly adjust their saccade size according to the rightward target displacement. Analyses on the mean biases (Fig. 1h) confirmed this relationship revealing a strong and significant difference in the amount of saccade adaptation between the two groups (t(26) = 3.14, *p* = 0.004, log_10_BF = 0.99). We then compared the effects of target shifting on visual perception between our subsample of participants. As for adaptive saccades, the transfer of the shift from motor to visual localization varied according to the personality traits of our sample (Fig. 1i, r = − 0.44, *p* = 0.01, log_10_BF = 0.37). Indeed, when comparing the mean adaptation effects (t(26) = 4.47, *p* < 0.001, log_10_BF = 2.3), we found a weaker adaptation effect of the shift from motor to visual localization for our high AQ sample (t(12) = − 2.45, *p* = 0.05, log_10_BF = 0.4), which instead was positive and significant only for the low AQ sample (t(14) = 3.89, *p* = 0.001, log_10_BF = 1.4). (Fig. 1j).

**Figure 2:**
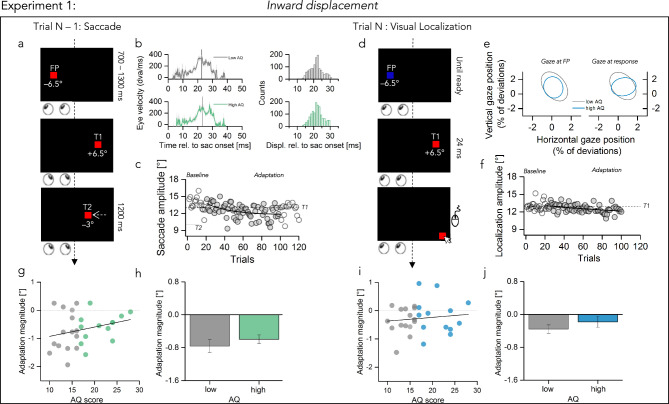
Experimental paradigm and main results of inward adaptation condition (exp1). (**a**) Schematic illustration of a saccade trial for inward adaptation condition. The procedure was the same as in Fig. 1a. However, here T1 was displaced 3° to its left (T2, inward displacement). (**b**) Averaged eye velocity profiles representing saccade performance in the saccade trials for low AQ and high AQ. Same as in Fig. 1b. (**c**) Saccade amplitudes over the course of the session for one example participant. Amplitudes decrease over the course of the trials in the direction of the target displacement (T2, black arrow at 10°). (**d**) Schematic illustration of a visual localization trial. Same as in Fig. 1d. (**e**) Variability of gaze position, measured as the Bivariate Confidence Ellipse (95% CI) of horizontal and vertical gaze position samples (percentage of imaged area). Same convention as in Fig. 1e. (**f**) Localization amplitudes over the course of the session for one example participant. Amplitudes decrease over the course of the trials following the saccade amplitudes trend. Dashed line at 13° indicate the location of T1. (**g**) Adaptation magnitudes in degree as a function of autism severity. Dashed line at 0 indicate no effect off target displacement, negative values indicate a shift of the effect in the direction of the displacement. Thick black line shows the linear fit through the data. (**h**) Adaptation magnitude averaged over the two groups of participants (low AQ gray, high AQ green). Bars represent average across participants, error bars show 1 SEM. (**i**,**j**) Same as (**g**,**h**) for visual localization data, color coded as gray for low AQ and light blue for high AQ.

In a different session, we asked participant to perform rightward saccades while the target was displaced to the left from the initial target position, resulting in an inward target displacement. The trial structure was the same of before: a saccade trial, in which the target jumped 3° inward, was always followed by a localization trial, in which the observer was asked to judge the position of a briefly flashed target by keeping the gaze on the fixation point (Fig. 2a,d). Displacements of the target were presented well within the period of saccade execution: on average 22.01 ms (± 1.92) after the saccade onset for low autistic traits—average saccade duration approximatively 40.41 ms (± 4.62)—and 22.83 ms (± 1.88) for high autistic traits—average saccade duration approximatively 38.9 ms (± 3.6) (Fig. 2b). As before, we ensured that only trials in which the localization was performed while keeping the gaze on the fixation point (not deviating more than 2.5° from fixation, see also method section) were used for analyses (Fig. 2e), and calculating the dispersion of horizontal and vertical eye position around the fixation point. As for outward adaptation session, there was no difference between our samples of participants in eye position variability nor during fixation (t(26) = − 1.08, *p* = 0.29, log10BF = − 0.27) nor during response (t(26) = − 1.64, *p* = 0.11, log_10_BF = − 0.02). As well as we didn’t find any differences in the absolute gaze positions between the two groups, either before saccade execution (gaze at fixation mean ± 1 SEM: low AQ = 0.27(0.17), high AQ = 0.315(0.12), t(26) = 0.49, *p* = 0.62, log_10_BF = − 0.65) or during response period (gaze at response mean ± 1 SEM: low AQ = 0.88(0.38), high AQ = 0.57(0.15), t(26) = 0.65, *p* = 0.52, log_10_BF = − 0.61).

We then plotted the time course of the session separately for saccade (Fig. 2c) and localization trials (Fig. 2f): displacing the target 3° inward led to modification in saccade amplitudes in the direction of the shift (see arrow, T2). Localization trials followed this amplitude shortening. Data for both trial types outside the baseline range were fitted with an exponential function which best described the decrease in amplitude over the course of the experiment (saccade trials Fig. 2c: $y=11.7+4.45{e}^{-0.04x}$; localization trials Fig. 2f: $y=10.6+2.79{e}^{-0.005x}$)

Results on the mean saccade adaptation and subsequent visual perception showed that when the target was stepped 3° to its left, the amount of saccade adaptation (Fig. 2g) and visual mis-localization (Fig. 2i) did not vary with the autistic symptomatology (saccade: r = 0.26, *p* = 0.17, log_10_BF = − 0.45, localization: r = 0.11, *p* = 0.55, log_10_BF = − 0.76). A two sample *t* test confirmed that displacing the target 3° inward led to a similar amount of saccade adaptation in both groups (Fig. 2h, t(26) = − 0.71; *p* = 0.48, log_10_BF = − 0.6) as well as a similar transfer of the adaptation effects to visual space (Fig. 2j, t(26) = –0.85; *p* = 0.4, log_10_BF = − 0.5).

**Figure 3:**
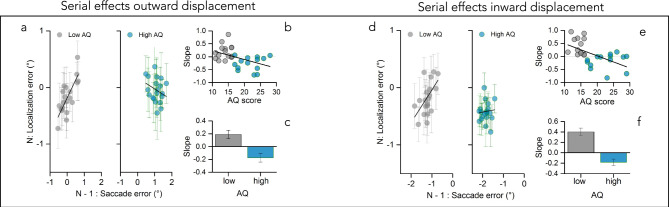
Serial effects between post-saccadic error and visual space for outward and inward displacement. (**a**) Average visual localization error (difference between target and perceived visual location) in trial N as a function of saccade error (difference between target position and saccade landing) in trial N–1 during outward displacement session for low AQ participants in gray (left panel) and high AQ participants in light blue (right panel). Black tick line represents linear fit through the data, from which the slope was extracted. Positive numbers on the x axis represent rightward displacements of the saccade target. Negative numbers on the y axis indicate localization error in foveal direction. The positive slope of the regression reveals that positive post-saccadic errors in trial N–1 led participants to localize the target further in the periphery in trial N. Error bars represent 1 SEM. (**b**) Slopes, representing the strength of serial dependency, for each participant as a function of autistic traits color coded as in (**a**). Thick black line shows the linear fit through the data. (**c**) Slope values averaged over the two groups of participants (low AQ gray, high AQ blue). Bars represent average across participants, error bars show 1 SEM. (**d**) Average visual localization error in trial N as a function of saccade error in trial N–1 during inward displacement session for low AQ participants in gray (left panel) and high AQ participants in light blue (right panel). Negative numbers on the x axis represent leftward displacements of the saccade target. Negative numbers on the y axis indicate localization error in foveal direction. Black tick line represents linear fit through the data, from which the slope was extracted. (**e**,**f**) same as (**b**,**c**).

We next sought to analyze the relationship between post-saccadic error and localization error. We estimated a linear regression between the position at which participant localize a target with a mouse pointer in trial n and the size of the post-saccadic error in trial n − 1 for each participant, separately for inward and outward displacements. Figure 3a shows the average visual localization as a function of errors in the final saccade landing position for one example participant with low symptomatology and one example participant with high autistic symptomatology, during the outward adaptation session. The positive slope of the regression reveals that positive post-saccadic errors in trial n − 1 led participants to localize the target further in the periphery in trial n. We used the slopes of the linear regressions to quantify the strength of serial dependence. We found a negative relationship between autistic traits and strength of serial dependency, which was trending according to a linear regression (Fig. 3b r = − 0.42, *p* = 0.02, log_10_BF = 0.2). We also observed that low autistic traits led to an attractive serial effect (t(14) = 2.61, *p* = 0.02, log_10_BF = 0.5), while high autistic symptomatology did not (t(12) = − 0.81, *p* = 0.05, log_10_BF = 0.9), with a significant difference between the two samples (Fig. 3c, t(26) = 4.14, *p* < 0.001, log_10_BF = 1.97). Moreover, a similar pattern of results was observed when the target was consistently shifted 3° inward from its initial position (Fig. 3d). As for outward displacements, the slopes of the linear regression between the position at which participants localized a target with a mouse pointer in trial n and the size and direction of the post-saccadic error in trial n − 1, were correlated with autistic traits (Fig. 3e). The correlation is negative, strong, and significant (r = − 0.60, *p* = 0.002, log_10_BF = 1.5), showing that the strength of the effect decreases with AQ. A two-sample *t* test (Fig. 3f) on the mean slopes of the two group confirmed this difference (t(26) = 4.84, *p* < 0.0001, log_10_BF = 2.65). In other words, visual localization judgments of participants with low AQ were attractively biased by the post-saccadic error, i.e., the difference between saccade landing and final saccade target position, while high AQ traits were associated with a weaker reliance of the past on the current sensory evidence (hence not adjusting their visual localization to the direction of the post-saccadic error).

These findings reveal an oculomotor inflexibility in participants with high autistic traits in trials in which changes in the target representation have to be applied. Two general reasons for these results are possible. First, they might solve the credit assignment problem different than low traits participants by interpreting the post-saccadic error as motion in the external world and therefore refrain from adapting the target coordinates. Second, an abnormal variance in the efference copy signal might impede the interpretation of the post-saccadic error and prevent adaptation. To disentangle between those two possibilities, we performed a second experiment in which the saccade target has to be localized across the execution of a saccade. Because vision is suppressed during saccades, observers will not perceive the displacement directly: correct localization depends on a comparison of the position before and after the saccades.

**Figure 4:**
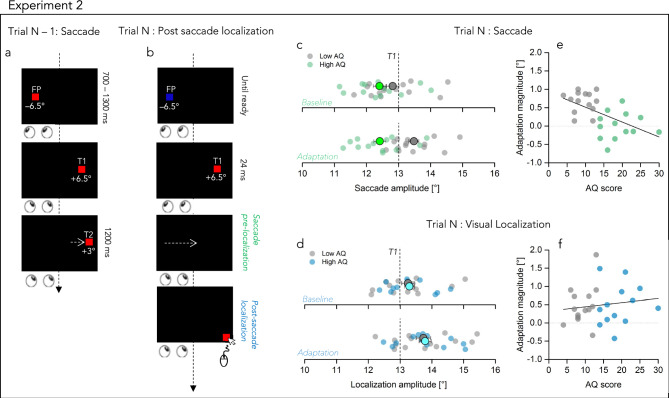
Experimental paradigm and main results of post-saccadic target localization (exp2). (**a**) Schematic illustration of a saccade trial in experiment 2. Same as Fig. 1a. (**b**) Schematic illustration of a visual localization trial in the post saccadic localization condition. Participants had to saccade (Saccade pre-localization) to the invisible target location and then localize (Post-saccade localization) the remembered target location, by using a mouse cursor which appeared at the very down right border of the screen resembling the target. (**c**) Averaged saccade amplitudes during baseline (upper panel) and adaptation (lower panel) for saccades over the course of the “post saccade localization” session. Each data point represents the mean of participants saccade amplitudes (low AQ in light gray, high AQ in light green). Dark gray and neon green point represents the mean over the two groups. Error bars are the standard error of the mean. Thin dashed line represents the location of T1. (**d**) Averaged localization amplitudes during baseline (upper panel) and adaptation (lower panel) over the course of the “post saccade localization” session. Each data point represents the mean of participants localization amplitudes (low AQ in light gray, high AQ in light blue). Dark gray and cyan points represent the mean over the two groups. Error bars are the standard error of the mean. Thin dashed line represents the location of T1. Localization amplitudes changed from baseline in the direction of the saccade outward shift for both groups of participants. (**e**) Adaptation magnitudes in degree as a function of autism severity for saccade trials. Adaptation magnitudes was calculated by subtracting the first trials of baseline from adaptation trials and then averaged across participants. Each data point represents one participant, low AQ in gray and high AQ in green. Dashed line at 0 indicate no effect off target displacement, positive values indicate a shift of the effect in the direction of the displacement. Thick black line shows the linear fit through the data. (**f**) same as in (**e**) for localization post saccade data. Solid black line is a linear fit passing through the data.

Twenty-seven additional participants were tested both on a post-saccadic target localization task and on a localization under fixation task, as in experiment 1. Again, the experiment contained saccade and localization trials. In the saccade trials participants were requested to perform a saccade to a target displaced 3° outward its initial position (Fig. 4a). Then, a localization trial started. In one session, a briefly flashed target had to be localized by mouse clicking while keeping fixation the fixation target. In a separate session, a briefly flashed target had to be localized by mouse clicking after a saccade was performed to the perceived target position in the periphery (see Fig. 4b). Hence, participants performed the subsequent localization judgment without post-saccadic visual references. We hypothesized that the efference copy generated before the saccade execution would help in the target localization by remapping the pre-saccadic target location and thus correctly localize the pre-saccadic target. Abnormal efference copies, instead, would result in a failure to remap the pre-saccadic target location and may lead observer to rely on the post saccadic eye position as a proxy for the subsequent localization.

Analyses on participants’ performance in the “localization under fixation” session (not shown as a figure), confirmed the results of outward displacement in experiment 1. The amount of saccade adaptation and its subsequent transfer to vision under fixation was altered in the high Autistic subsample. While participants scoring low on the questionnaire increased their saccade amplitudes compared to baseline level, resulting in a positive and significant effect of adaptation (t(13) = 11.47, *p* < 0.001, log_10_BF = 5.5), the high AQ subsample did not show any changes in saccade amplitudes (t(12) = − 1.26, *p* = 0.23, log_10_BF = − 0.3). Moreover, results showed that the effect of saccade adaptation were again decreasing as a function of Autistic Traits (r = − 0.84, *p* < 0.001, log_10_BF = 5.83). Accompanying changes in visual localization after saccade adaptation were following a similar trend: changes in localization amplitudes from baseline were negatively correlated with the symptoms severity (r = − 0.39, *p* = 0.04, log_10_BF = 0.03) and resulted in a significantly different amount of adaptation between the two groups (t(25) = 2.82, *p* = 0.009, log_10_BF = 0.73).

We then moved to analyze the data in which a saccade target has to be localized across the execution of a saccade. Here a saccade trial, which had the purpose of inducing motor adaptation (see Fig. 4a, Trial N − 1: Saccade) was followed by a localization post-saccade trial (Fig. 4b, Trial N: Post saccade localization).

Figure 4c,d shows the mean saccade amplitudes for each participant during the first 20 valid trials of baseline and the adaptation trials, for saccades pre localization (in gray for low AQ and green for high AQ) and post-saccadic localization (in gray for low AQ and light blue for high AQ). In the baseline phase, saccades of both groups slightly undershot the target. After this phase, endpoints shifted rightward in the direction of the target displacements only for the low AQ subsample. The extent of the adaptive shift was measured by the distance between the pre-adapted and adapted saccade endpoints as well as the pre-adapted and adapted localization reports. We first compared the effects of target shifting on saccades between our subsample of participants. Results confirmed our previous outward adaptation experiment’s finding: while participants with low AQ learned to lengthen their saccade amplitudes over time in the direction of the target displacement (on average ~ 0.7°, t(13) = 8.6, *p* < 0.001, log_10_BF = 4.2), the high AQ subsample failed to remap the pre-saccadic target position to its predicted target location (on average ~ 0°, t(12) = 0.1, *p* = 0.93, log_10_BF = − 0.6). Figure 4e plots the relation between saccade pre-localization changes and the autistic symptomatology. Results reveal a positive and significant correlation, showing once again that motor adaptation decreases as the autistic traits increase (r = − 0.55, *p* = 0.002, log_10_BF = 1.13). Moreover, in post saccade localization trials in which the target had to be localize after performing a saccade to the remembered (and invisible) target location, localization judgments during the adaptation phase for low AQ shifted rightward relative to pre-adaptation (Fig. 4f, significantly different from 0, Student’s *t* test, *p* < 0.05), as much as in the previous experiment. Together with them, also the high AQ subsample surprisingly showed a visual mis-localization in the direction of the visual target: their perceptual judgements shifted from 13.29° in the pre-adaptation phase to 13.79° in adaptation. That is to say, although the high autistic phenotype did not adapt their motor map to the perceptual change, their visual map changed significantly in the adaptation phase (significantly different from 0, *p* = 0.008) and, moreover, in much the same manner as in the lower AQ phenotype (t(25) = − 0.06, *p* = 0.95, log_10_BF = − 0.44), confirmed also by a not significant correlation with the autistic symptomatology (Fig. 4f, r = 0.13; *p* = 0.5, BF = − 0.73).

## Discussion

In this study, we investigated sensory and motor behaviors over a wide range of autistic symptomatology. We first focused on adaptive saccade flexibility and the consequences on the perception of space. We found a negative anecdotal relationship between autistic traits and adaptation strength in saccade outward gain shifts, which was however confirmed by a strong group difference (LogBF = 2.3). In these sessions, participants with high autistic traits did not adapt to the post-saccadic error. However, no such modulation of adaptation strength by autistic traits was found for inward adaptation, although the evidence in favor of our results were less strong than expected (|Log_10_BF| = 0.45).

Different neural processes control adaptive gain decrease and increase (see^20^ for a review). Whereas for gain increase target coordinates of saccade control are shifted, gain decrease might occur at the level of the motor plan^20,34,36,55,56^.

Persistence of movement vectors despite motor errors might result from biased inferences in the credit assignment^11^. If motor errors are not interpreted as such, then no stimulus is driving the sensorimotor system to invest into movement gain change. A clear prediction follows from this hypothesis: Saccades of participants with high autistic symptomatology should remain unadapted after experiencing intra-saccadic target displacements in outward direction, but their perceptual judgements of where they believe the target to be located should be shifted in the direction of the target displacement. In order to localize the target across a saccade, information about the size of the gaze shift is necessary if no visual landmarks are available. The size of the gaze shift is encoded in the efference copy^57^. If this signal is uninformed of the saccade gain change, mis-localization of the target must ensue^38,43,58^. In high AQ participants, mis-localization in the same direction, despite the absence of adaptation, would be expected if they attribute post-saccadic errors to a target motion on the screen. We conducted a second experiment to test these predictions. Before and after gain outward adaptation, we asked participants to localize the saccade target after they completed their saccades. The saccade target disappeared during execution of the saccade. Participants with low autistic traits adapted saccades in this task and mis-localized the target outward. In line with the results of experiment 1, we found that the gain in saccade adaptation depended on the autistic symptomatology, becoming weaker for people with higher autistic traits. However, although they landed on the primary target position, they mis-localized the saccade target outward in direction of the secondary, i.e., displayed target position. Since participants with high autistic traits landed on the primary target correctly, no adaptive shift of visual space nor an efference copy uninformed of adaptation can explain the mis-localization. It is worth noting that no target jumps were occurring during a localization trial, hence recalibration of the visual map was provided by the size of the post-saccadic error from previous trials. In this view it is possible that in high AQ participants the sensorimotor system registered the post-saccadic error from previous trials but inferred that it came from an unstable moving world (in this case the target jumping) rather than their own motor accuracy. Altered causal inference about motor errors might explain the autistic tendency to rely more on the sensory evidence (the target has jumped) than on their own (inaccurate) motor actions. Previous research suggests that both autistic individuals and non-autistic individuals with a higher number of autistic traits rely less on prior knowledge when interpreting the world^59–61^ resulting in weaker priors in response to social^62–64^ as well as non-social social stimuli presentation^63,65–67^.

Following this reasoning, we have analyzed our data to estimate the strength with which participants took into account the error information of the preceding trial. After a saccade is completed, the sensorimotor system registers the position of the eyes on the target and any deviation between the eye landing position and the actual target is the post-saccadic error. This error will shape the amplitude of the following saccade such as to re-establish the predicted saccade landing. Many studies have demonstrated that the modification of motor variables by saccade adaptation leads to a recalibration of visual localization of the target executed by hand pointing or by perceptual reports^40–42^. More recently, we have shown the post-saccadic errors recalibrate visual space in a serial dependency manner^47^. In the present study we found that judgments of participants with high AQ traits were associated with a weaker reliance of the past on the current sensory evidence, i.e. their visual space was not updated by the post-saccadic error information. In line with these findings, previous studies have shown that autistic people show a reduced ability to learn from motor errors, suggesting poor saccade control and learning processes which reflected reduced plasticity in the oculomotor vermis circuits of the cerebellum^68^. Moreover, other studies have reported abnormal eye movement dynamics^69,70^, reduced saccadic accuracy^71,72^, larger trial-to-trial variability of saccade accuracy and amplitude^69,72^ and preserved saccade velocity, latency^69,73,74^ and precision^71^ in ASD. Importantly, abnormal oculomotor behavior seems to appear early in life in ASD and often precedes the emergence of social communication and high-level cognitive deficits^71^.

In conclusion, our data suggest that a biased inference in understanding their own action in high autistic symptomatology might prevent oculomotor learning. These results might imply that each saccade high autistic traits produce might be accompanied by slight perceptual disturbances. Consequently, oculomotor inflexibility would increase the perceptual bombardment that is experienced in ASD.

## Materials and methods

### Participants

All participants were recruited through the Heinrich-Heine University Düsseldorf and received either course credit or payment of 10 euros/h. Experimental procedures were approved by the local ethics committee of the psychological department of the Heinrich-Heine—University Düsseldorf. Written informed consent was obtained prior to the experiment in accordance with the declaration of Helsinki. Twenty-eight participants naive to the purpose participated in experiment 1 (19 females, age and std = 21.8 ± 2.6) and 27 different participants than in Experiment 1 and naive to the purpose of the study took part in experiment 2 (13 females, age and std = 22.01 ± 3.87).

### AQ scores

All participants completed the self-administered Autistic Quotient questionnaire, in either the validated English or German version^48,49^. This contains 50 items, grouped in five subscales: attention switching, attention to detail, imagination, communication and social skills. For each question, participants read a statement and selected the degree to which the statement best described them: “strongly agree,” “slightly agree,” “slightly disagree,” and “strongly disagree”. The standard scoring described in the original paper was used: 1 when the participant’s response was characteristic of ASD (slightly or strongly), 0 otherwise. Total scores ranged between 0 and 50, with higher scores indicating higher degrees of autistic traits. All participants scored below 32, the threshold above which a clinical assessment is recommended^50^.

As we were interested in the effect of autistic personality traits on the results, we divided participants into low and high Autistic Quotients, based on a median split of their AQ scores. The median of the scores was 17 (with lower and upper quartiles of 14 and 24) for experiment 1 and 13.5 for experiment 2 (with lower and upper quartiles of 10 and 18). Scores of both experiments were normally distributed, as measured by the Jarque–Bera goodness-of-fit test of composite normality (exp1: JB = 2.09, *p* = 0.18; exp2: JB = 4.69, *p* = 0.15).

### Apparatus

Participants were placed at a 57 cm distance from a CRT monitor (12.9 inches, Diamond Pro 2070). The visible screen diagonal was 41.5 cm, resulting in a visual field of 40° × 30°. To avoid visual references the room was completely dark. A transparent foil reduced the luminance of the monitor by 2 log units and prevented the visibility of the monitor borders. All stimuli were presented with a refresh rate of 120 Hz and a resolution of 800 × 600 pixels.

### Stimuli and procedure

Stimuli were generated under Matlab 2006b (v. 7.10.0; The MathWorks, Natick, MA, United States) using PsychToolbox routines (v. 3.0.17^75^) run by a Macintosh computer (MacMini, 2014) and presented on an external screen displaying a uniform black background (0.01 cd/m^2^). All stimuli were red or blue squares (∅: 0.55° × 0.55°). Each session started with the presentation of a fixation square, at the vertical meridian, 6.5° to the left of the screen center, to which participants had to direct their gaze. The color of the fixation square alternated according to whether the participant was asked to move its eyes or to fixate: a red fixation square signaled that a rightward saccade had to be executed to a saccade target, and a blue square indicated to keep fixation thorough the entire duration of the trial (in experiment 1) or to saccade to a red target extinguished before saccade ended (experiment 2).

In experiment 1, a saccade trial started with the presentation of the fixation square. After 700–1300 ms, the fixation square disappeared and a saccade target (0.55° × 0.55°, red color) was presented 6.5° to the right of the center of the screen (T1). When the eye-tracker detected the initiation of a saccade (eye velocity exceeding 30°/s), the saccade target was displaced + 3° to the right or − 3° to the left of its initial position (T2), depending on the condition tested (outward or inward adaptation, respectively). Participants were instructed to make a saccade to the target location and to stay fixated at the saccade landing location. The saccade target disappeared after 1200 ms and a localization trial started automatically (see Fig. 1a for an example of a trial sequence). In a localization trial, participants re-directed the gaze at the fixation target location. The FP (0.55° × 0.55°, blue color) was presented 6.5° to the left of the screen center, as for the saccade FP. When ready, participants pressed the space bar on the keyboard to start a trial. A red square (analogous to the saccade T1) was flashed for 24 ms after fixation point offset. A mouse pointer (square 0.55° × 0.55°, red color) was presented in the right lower border of the screen. Participants had to click the cursor at the perceived flashed position (or at the lowest position possible if they had not perceived the flash) while remaining fixated at the invisible fixation point location. As the fixation point turned off before flash onset and the cursor appeared after flash offset, there were no visual references that could affect the perceived flash position. In experiment 2, a saccade trial was identical to an outward saccade trial in exp 1: an eye movement was performed, after fixation extinction, to a target which was shifted 3° outward its initial position (Fig. 4a). A localization trial started with a blue fixation square as in experiment 1. Session 1 was the same as experiment 1: a saccade trial was followed by a localization trial under fixation. In session 2, however, participants were instructed to aim their gaze at the briefly flashed target as fast and as accurately as possible once it was extinguished, and to stay fixated in the dark at the saccade landing location (post-saccadic localization trials, Fig. 4b). As in experiment 1, participants clicked the cursor at the perceived flash position while still fixating at the saccade landing location.

Participants completed one session of each condition (2 sessions for experiment 1: inward adaptation, outward adaptation; and 2 sessions for experiment 2: localization under fixation and localization post-saccadic), which comprised of 100 trials of saccade and 100 trials of localization. The order of the conditions was pseudo-randomized within and between participants.

### Eye movements and data analysis

Eye movements and pupil diameters were recorded with the EyeLink 1000 system (SR Research Ltd., Mississauga, Ontario, Canada), which sampled eye positions at a rate of 1000 Hz. The head was sustained with a chin- and forehead-rest. For all participants the left eye was recorded. Viewing was binocular. At the beginning of each session, the Eyelink was calibrated with the standard nine-point Eyelink procedure. The system detected the start and the end of a saccade when eye velocity exceeded or fell below 30°/s. In both adaptation conditions of exp 1 and in the saccade trials of exp 2, a trial was rejected if a saccade landing position was smaller than 3.5°, or if a blink was detected at the time of the saccade or if a saccade was initiated before the fixation target disappearance. Similarly, in exp 1 a localization trial was rejected if the mouse clicking position was smaller than 3.5°, or if participant shifted their gaze outside an invisible window with a diameter of 2.5 during the trial. A post saccadic localization trial (exp 2) was excluded if the mouse clicking position was smaller than 3.5° or if participant did not perform a saccade before the visual localization (that is if gaze deviated less than 2.5° from fixation) or if a saccade was executed before the target extinction.

The first 20 valid trials of experiment 1 and the first 20 of experiment 2, were considered as baseline and subtracted from the saccade amplitudes and visual localization, in order to calculate adaptation and localization effects. In all the analyses the raw saccade amplitudes and mouse localizations were used, except for the serial visuomotor dependences analyses in which the saccade and localization trials were averaged into 24 separate bins each containing 1/5th of the trials. To evaluate recovery from adaptation, participants completed 20 additional de-adaptation trials at the end of each adaptation condition (in which they made saccades to stimuli presented 13° from fixation in the same hemifield in which adaptation had been tested). Note that these last 20 raw trials, were mainly used to bring the participants back to their initial state of de-adaptation and therefore results from these trials were not included for statistical analyses.

For localization trials we checked for possible eye-movement patterns at fixation or during the mouse response that could have been different between our group of participants, which would have affected localization performance. We therefore measured the variability of eye position around fixation point on each trial for each participant. Specifically, the dispersion of eye position was calculated as the bivariate confidence ellipse area (BCEA), that is the area of a bivariate contour ellipse encompassing eye position samples, defined as:

1 $$BCEA=2*k*{\sigma }_{H}*{\sigma }_{V}*{[\left(1-\rho \right)]}^{0.5}$$

where k is the confidence limit for the ellipse, ${\sigma }_{H}$ and ${\sigma }_{V}$ are the standard deviation of eye positions in the horizontal and vertical meridian respectively, and $\rho$ is the product-moment correlation of these two position components. As in previous studies^76–78^, we set k = 1.14, thus the probability of a given observation falling within the ellipse was 68% ($1-e-k$). Figures 1e and 2e shows the dispersion of eye movements for the two groups of participants by pooling all the data together.

We fitted an exponential model to the data outside the baseline trials with three free parameters: the amplitude of the decay/growth (A), the decay/growth rate (R0), and the y offset (y0):

2 $$y=y0+A{e}^{R0x}$$

The sign of R0 could be negative or positive depending on the condition tested (positive for amplitudes lengthening and negative for shortening).

Our main analyses compared data across conditions and groups of participants: Standard *t* tests, and correlation analyses were complemented with Bayes factors estimation^79^. Bayes factors are reported in logarithmic base 10 units (log_10_BF) and their absolute values should be interpreted as providing anecdotal (0–0.5), substantial (0.5–1), strong (1–1.5), or very strong (> 1.5) evidence, in favor of the alternative hypothesis if positive or the null hypothesis if negative.

## Data Availability

Data are available on Zenodo at the link: https://zenodo.org/record/7840564.
